# Supplementary material for: Ranking the biases: The choice of OTUs vs. ASVs in 16S rRNA amplicon data analysis has stronger effects on diversity measures than rarefaction and OTU identity threshold
Source: PLoS One. 2022 Feb 24;17(2):e0264443. doi: 10.1371/journal.pone.0264443 (PMC8870492; doi:10.1371/journal.pone.0264443)
Supplement: S1 File — (DOCX) [file pone.0264443.s001.docx]

**SUPPLEMENTARY FIGURES**

**Ranking the biases: the choice of OTUs vs. ASVs in 16S rRNA amplicon data analysis has stronger effects on diversity measures than rarefaction and similarity threshold**

Marlène Chiarello^1*^, Mark McCauley^1^, Sébastien Villéger^2^ and Colin R Jackson^1^

^1^University of Mississippi, Department of Biology, University, MS, USA

^2^MARBEC, University of Montpellier, CNRS, Ifremer, IRD, Montpellier, France

*Corresponding author:

[**marlene.chiarello@gmail.com**](mailto:marlene.chiarello@gmail.com)


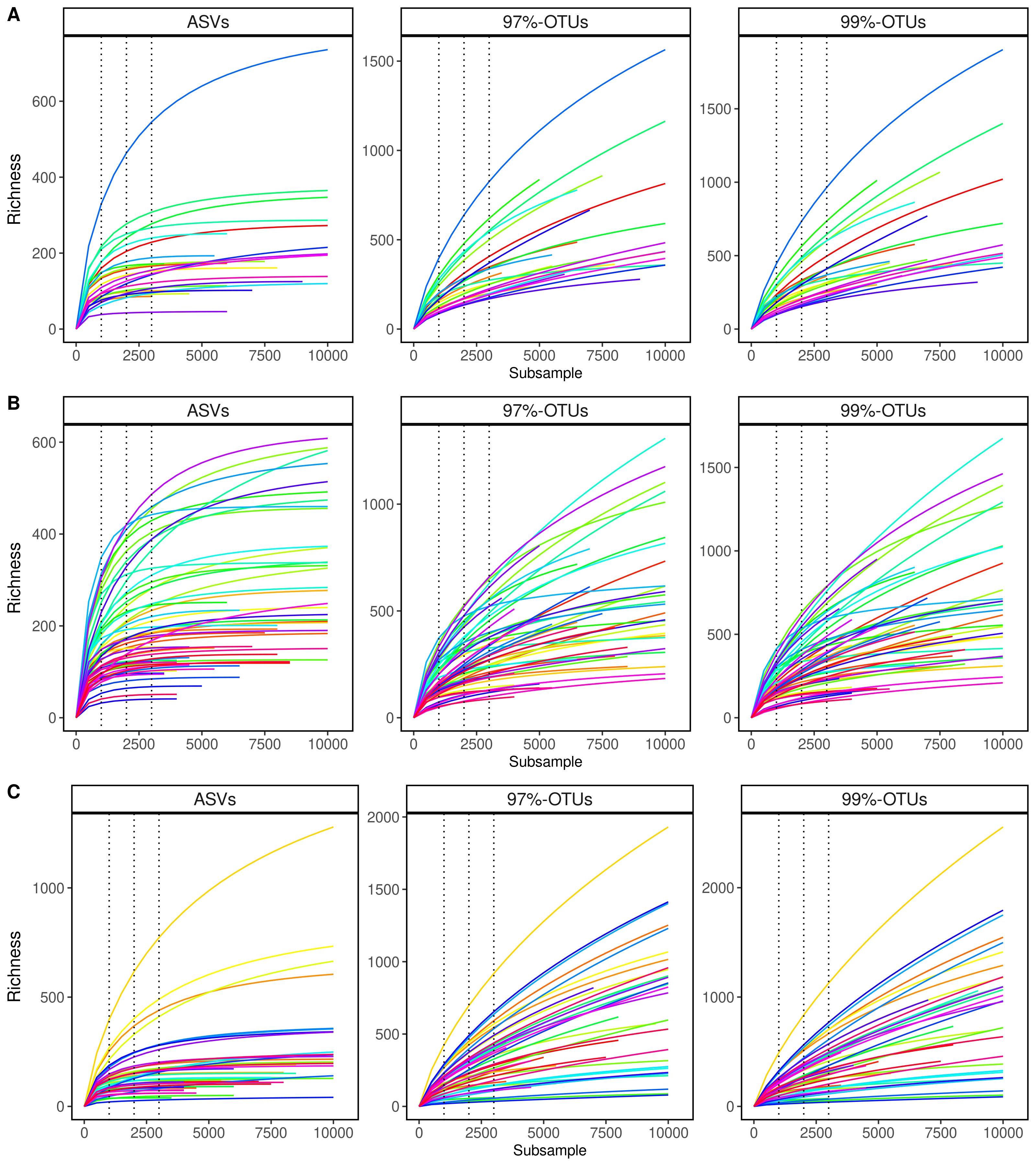


**S1 Fig: Rarefaction curves of mussel microbiome samples** obtained after sequence processing using DADA2 (ASVs) and Mothur pipelines (97 %- and 99 %-OTUs), before rarefaction. Rarefaction data was obtained by increasing random subsampling of 0 to 10,000 sequences on the unrarefied community table, and computing the number of operational units (Richness). Plots A, B and C represent microbiomes of *Amblema plicata*, *Cyclonaias asperata* and *Lampsilis ornata*, respectively. Random colors were assigned to samples to facilitate comparison between panels. The dotted lines represent the three rarefaction levels, *i.e.* 1,000; 2,000 and 3,000 sequences per sample.

**
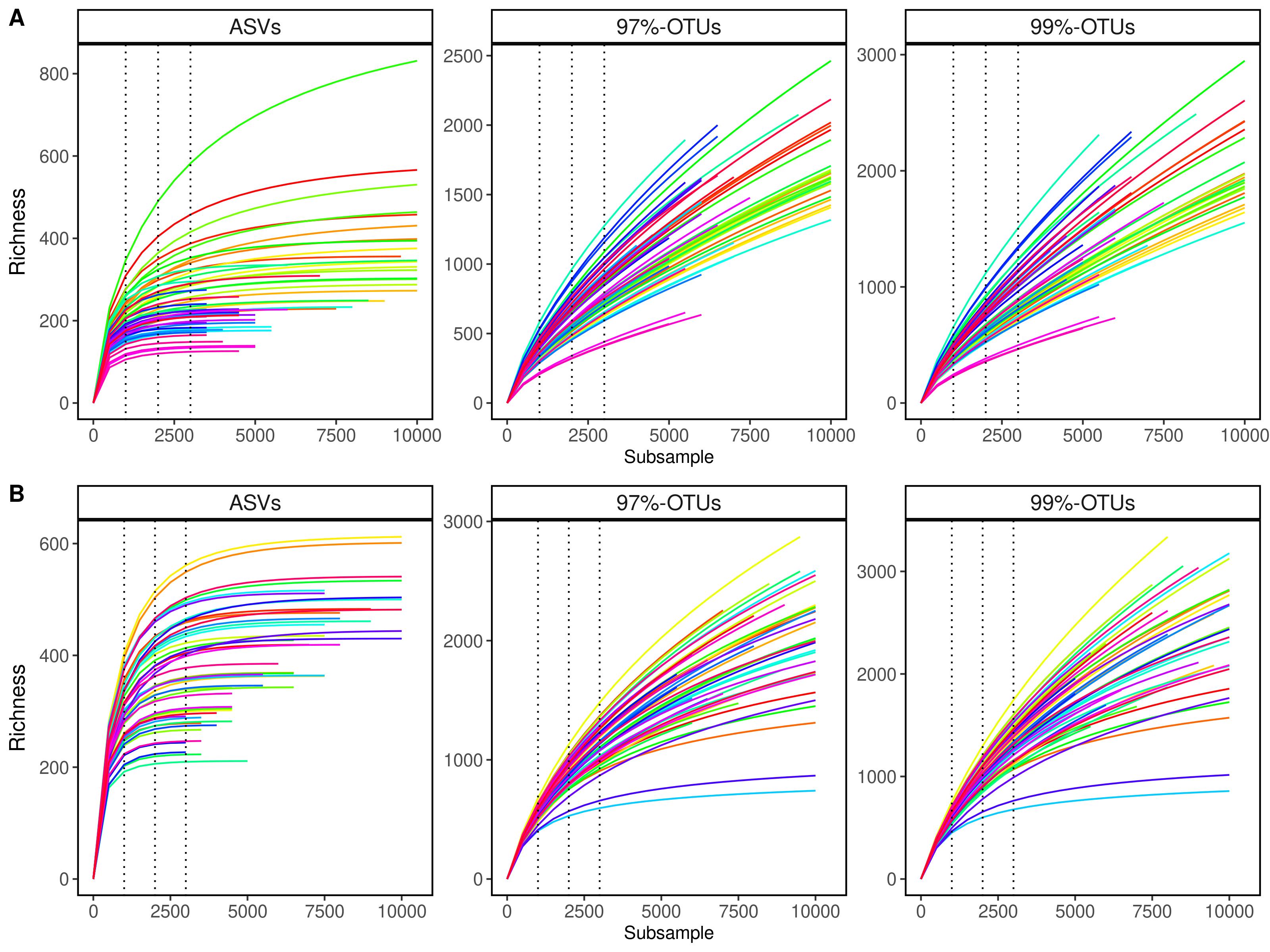
**

**S2 Fig: Rarefaction curves of seston (A) and sediment samples (B)** obtained after sequence processing using DADA2 (ASVs) and Mothur pipelines (97 %- and 99 %-OTUs), before rarefaction. Rarefaction data was obtained by increasing random subsampling of 0 to 10,000 sequences on the unrarefied community table, and computing the number of operational units (Richness). Random colors were assigned to samples to facilitate comparison between panels. The dotted lines represent the three rarefaction levels, *i.e.* 1,000; 2,000 and 3,000 sequences per sample.


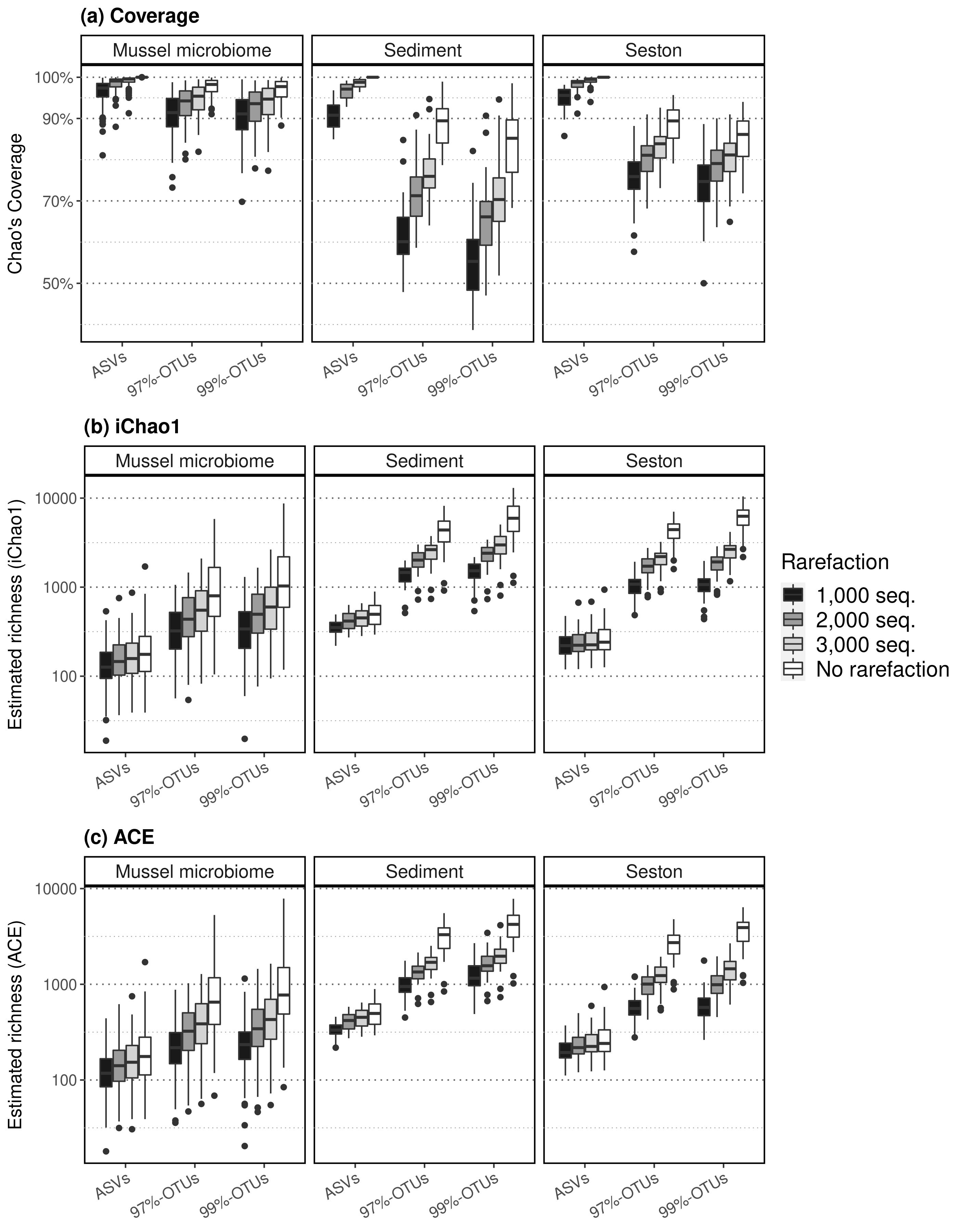
**S3 Fig: Effect of sequence processing methodology on sampling coverage and estimators of richness correcting for unobserved biological units**. For each community type (mussel gut microbiome, sediment, seston), estimators were computed before rarefaction and after rarefaction to 1,000; 2,000 and 3,000 sequences per sample. (a) Estimation of sampling coverage measured by Chao’s coverage estimator; (b and c) Total sampled + unobserved richness estimated within the samples, measured using (b) iChao1 and (c) ACE indices. To ease the visualisation of differences across methods and rarefaction levels, the y-axis of plots (b) and (c) has been log transformed.


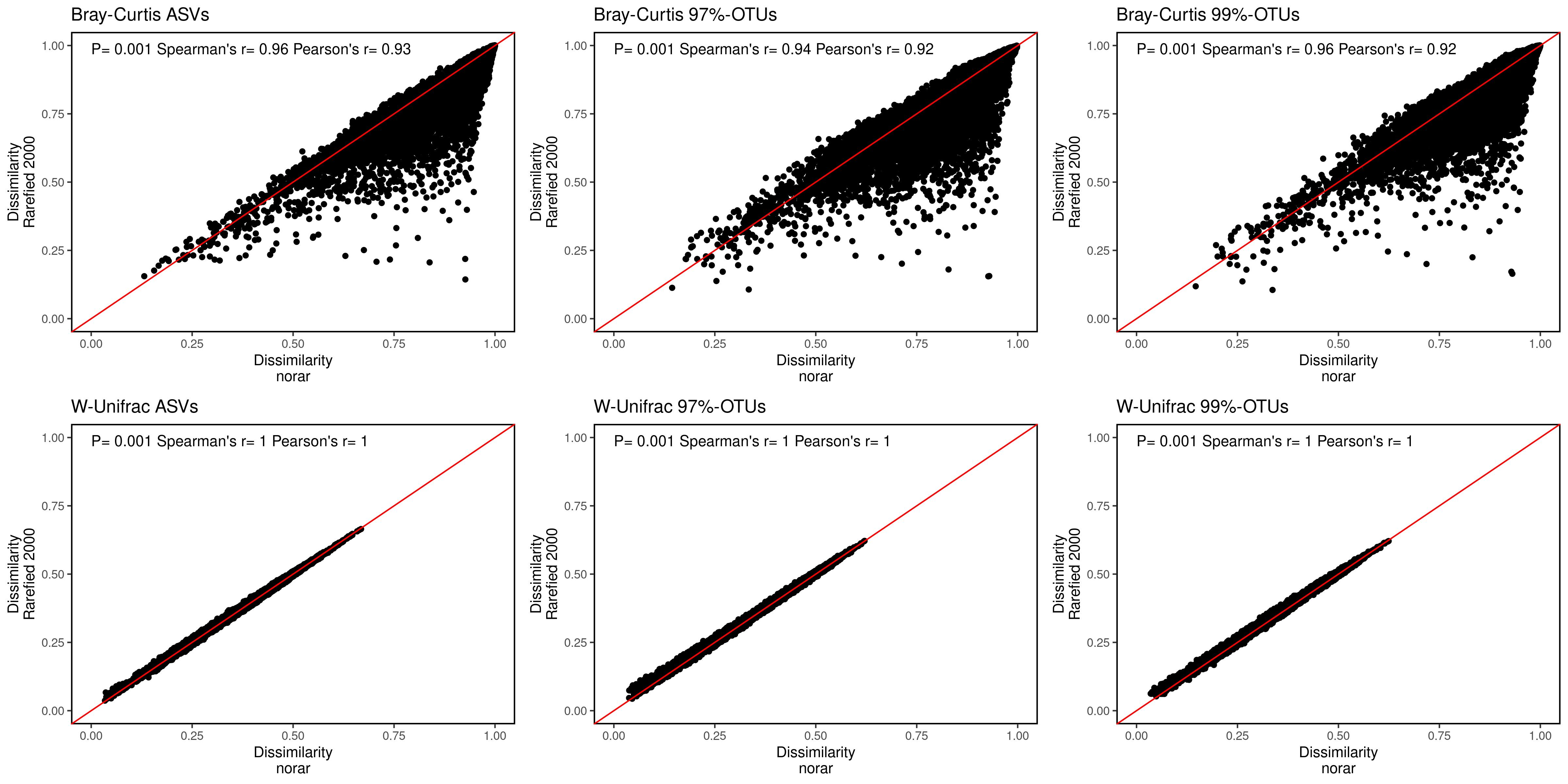
**S4 Fig: Change in Bray-Curtis and W-Unifrac beta diversity after rarefaction to 2,000 sequences per sample within the ASV-, 97%- and 99%-OTUs datasets across all sediment, seston and mussel microbiomes.** The agreement of ranks of beta diversities before and after rarefaction were assessed using a Mantel test, which result is indicated over each plot. The diagonal is marked by the red line. The same correlations on other beta diversity metrics (Jaccard and U-Unifrac) is available in Supplementary Figure 5.


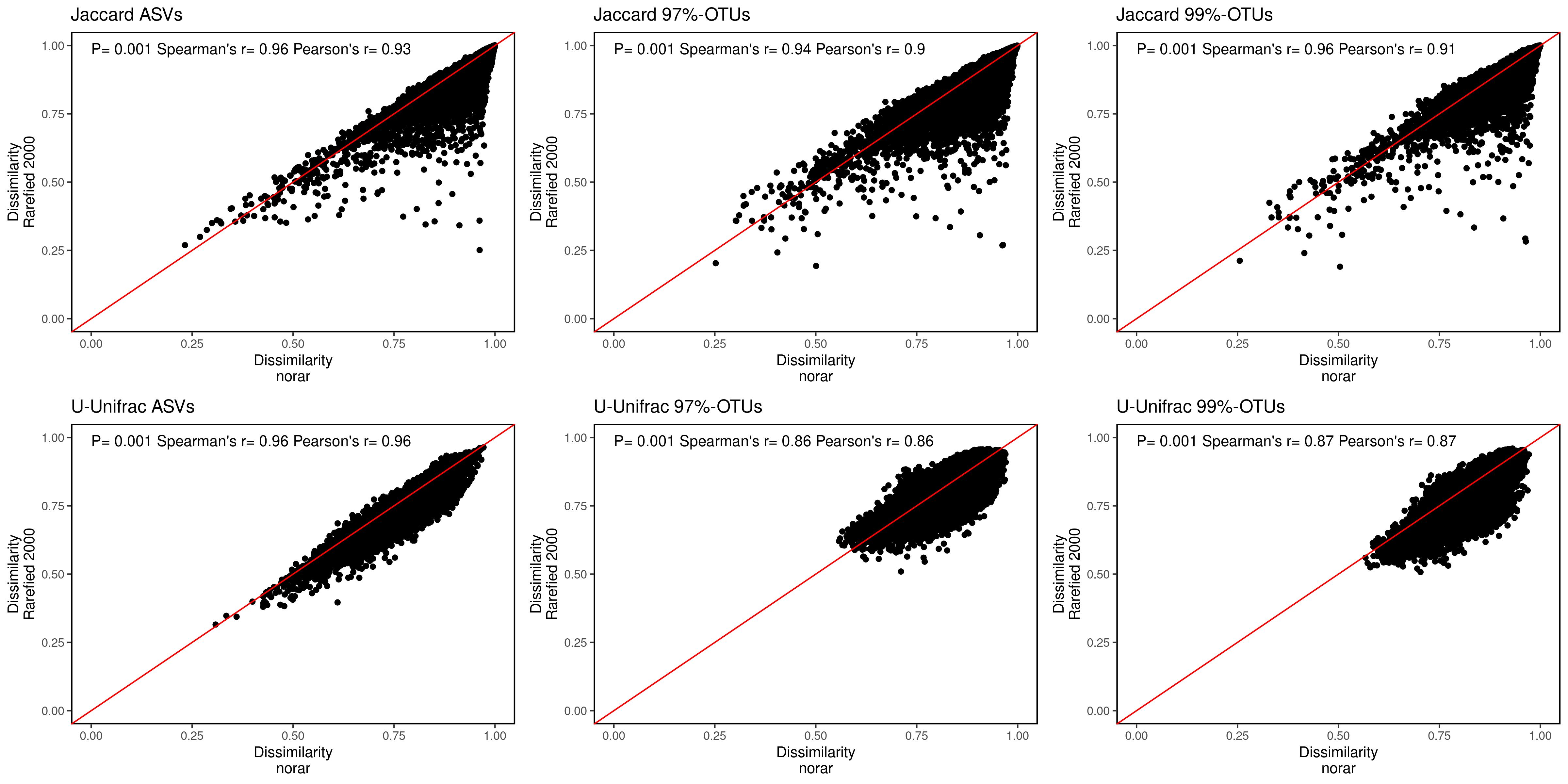
**S5 Fig: Change in Jaccard and U-Unifrac beta diversity after rarefaction to 2,000 sequences per sample within the ASV-, 97%- and 99%-OTUs datasets across all sediment, seston and mussel microbiomes.** The agreement of ranks of beta diversities before and after rarefaction were assessed using a Spearman’s correlation tests, which result is indicated over each plot. The diagonal is marked by the red line.


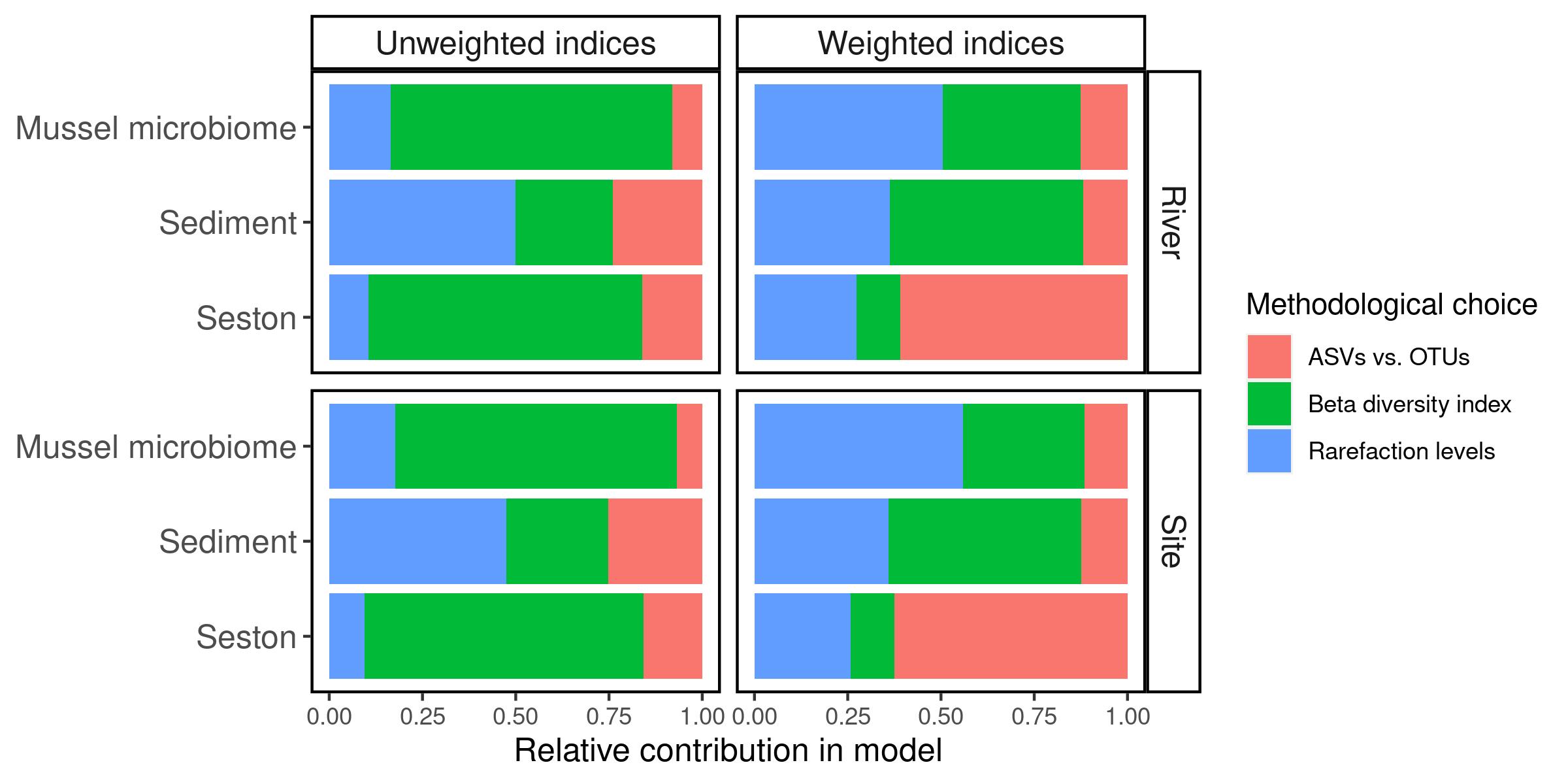
**S6 Fig: Relative effects of the index of the dissimilarity metric used, OTUs vs. ASVs methods, and the different levels of rarefaction, on the quality of detection of biological signal (River effect, top, and Site effect, bottom) by the PERMANOVAs.** The analysis was perfomed separately based on OTU or ASV relative abundance weighted and unweighted (presence/absence) indices, where the relative contribution of each methodological choice was assessed using separated random forest models.

Random forest models assessing the relative contribution of the same methodological choices on the intensity of the ‘community type’ signal based on unweighted indices gave a relative contribution of 60.7%, 21.0% and 18.3% for the choice of beta diversity index, OTUs vs. ASVs, and rarefaction, respectively. Based on weighted indices, those were 82.3%, 9.6% and 8.0%, respectively.

**
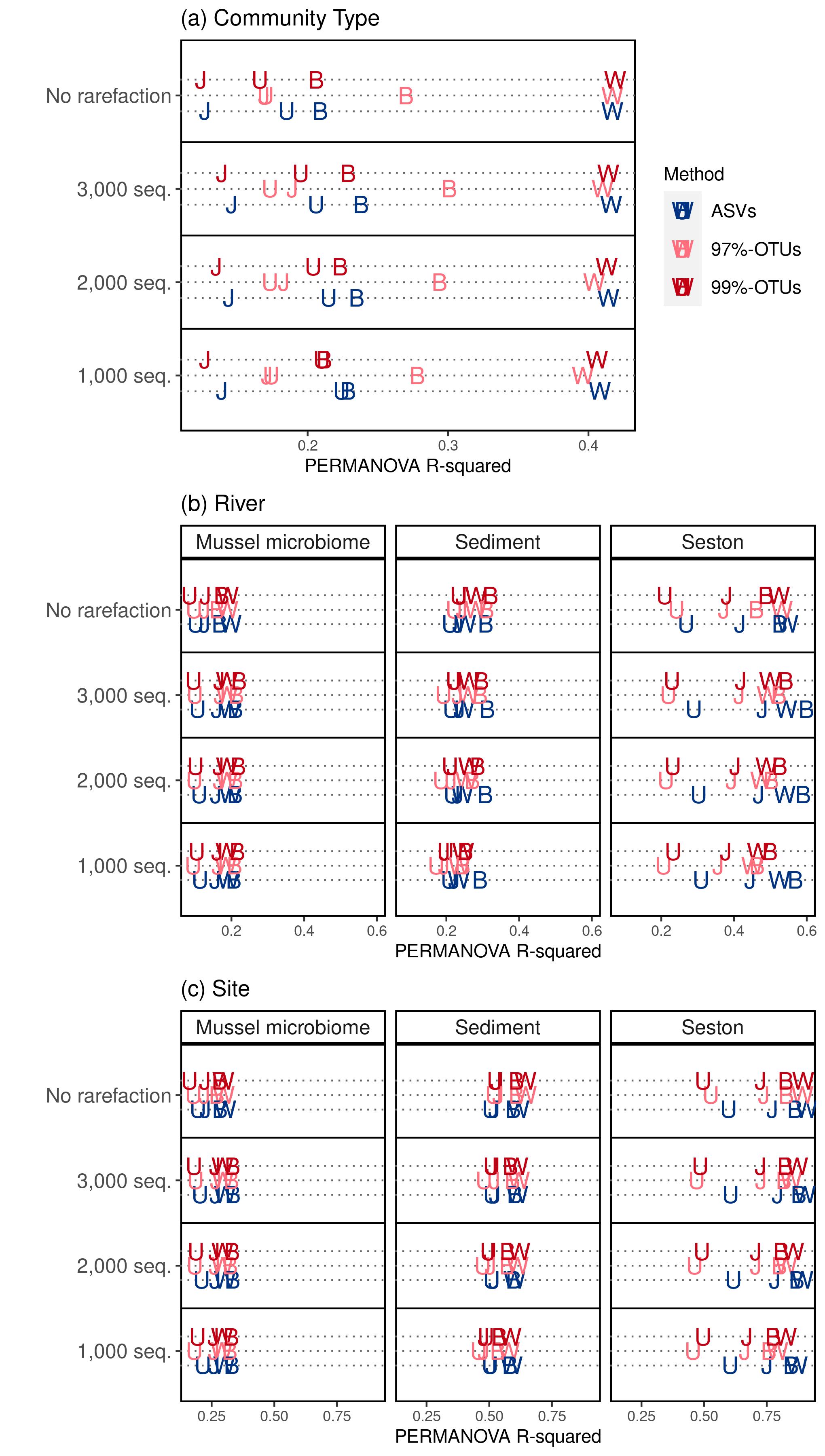
**

**S7 Fig: Quality of detection of biological signal across sequence processing methods, rarefaction levels and dissimilarity indices.** The intensity of the effect of (a) community type, (b) sampling river and (c) collection site on community structure was assessed using separated PERMANOVAs for each factor, and each combination of sequence processing method x rarefaction level x index of dissimilarity. U: U-Unifrac ; W: W-Unifrac ; B:Bray-Curtis ; J: Jaccard.
