# Supplementary material for: Ranking the biases: The choice of OTUs vs. ASVs in 16S rRNA amplicon data analysis has stronger effects on diversity measures than rarefaction and OTU identity threshold
Source: PLoS One. 2022 Feb 24;17(2):e0264443. doi: 10.1371/journal.pone.0264443 (PMC8870492; doi:10.1371/journal.pone.0264443)
Supplement: S2 File — (DOCX) [file pone.0264443.s002.docx]

**SUPPLEMENTARY TABLES**

**Ranking the biases: the choice of OTUs vs. ASVs in 16S rRNA amplicon data analysis has stronger effects on diversity measures than rarefaction and similarity threshold**

Marlène Chiarello^1*^, Mark McCauley^1^, Sébastien Villéger^2^ and Colin R Jackson^1^

^1^University of Mississippi, Department of Biology, University, MS, USA

^2^MARBEC, University of Montpellier, CNRS, Ifremer, IRD, Montpellier, France

*Corresponding author:

[**marlene.chiarello@gmail.com**](mailto:marlene.chiarello@gmail.com)

**S1 Table**: Number of samples (*n*) of river sediment, seston and microbiomes of three different species of freshwater mussel (*Lampsilis ornata*, *Amblema plicata* and *Cyclonaias asperata*) collected in six different rivers during summer 2019. The number of sampling sites (*n* sites) is also indicated.

|  |  | **Rivers** | | | | | | **Total** |
| --- | --- | --- | --- | --- | --- | --- | --- | --- |
|  |  | Bogue Chitto | Bear creek | Sipsey | Paint Rock | Buttahatchee | Duck |  |
| ***n* sites** | | 2 | 2 | 5 | 3 | 2 | 4 | **18** |
| Sample type | Sediment | 5 | 5 | 15 | 5 | 4 | 11 | **45** |
|  | Seston | 6 | 6 | 13 | 8 | 6 | 12 | **51** |
|  | *L. ornata* | 2 | 0 | 31 | 0 | 10 | 0 | **43** |
|  | *A. plicata* | 8 | 4 | 4 | 3 | 0 | 4 | **23** |
|  | *C. asperata* | 14 | 0 | 25 | 0 | 14 | 0 | **53** |
|  | Total Mussels | 24 | 4 | 60 | 5 | 24 | 4 | **121** |

**S2 Table:** Number of sequences before sequence processing (Nseq), and after sequence processing using both pipelines tested (Mothur at 97 % and 99 % OTU threshold, and DADA2) before rarefaction.

| **Sample** | **Site** | **River** | **Species** | **Type** | **Nseq** | **Nseq after processing** | | |
| --- | --- | --- | --- | --- | --- | --- | --- | --- |
|  |  |  |  |  |  | **ASVs** | **97%-OTUs** | **99%-OTUs** |
| MDB0015 | 1 | Bogue Chitto | *C. asperata* | Mussel | 10385 | 8554 | 5099 | 5089 |
| MDB0016 | 1 | Bogue Chitto | *C. asperata* | Mussel | 17467 | 13719 | 14942 | 14805 |
| MDB0017 | 1 | Bogue Chitto | *C. asperata* | Mussel | 9655 | 7900 | 8120 | 8105 |
| MDB0018 | 1 | Bogue Chitto | *C. asperata* | Mussel | 18742 | 15756 | 16668 | 16593 |
| MDB0019 | 1 | Bogue Chitto | *C. asperata* | Mussel | 10287 | 8170 | 8503 | 8490 |
| MDB0021 | 1 | Bogue Chitto | *C. asperata* | Mussel | 8282 | 5893 | 6548 | 6519 |
| MDB0023 | 1 | Bogue Chitto | *C. asperata* | Mussel | 20963 | 17158 | 12360 | 12325 |
| MDB0024 | 1 | Bogue Chitto | *C. asperata* | Mussel | 17742 | 14387 | 14852 | 14827 |
| MDB0036 | 1 | Bogue Chitto | *A. plicata* | Mussel | 23923 | 16360 | 17771 | 17697 |
| MDB0037 | 1 | Bogue Chitto | *A. plicata* | Mussel | 8515 | 6458 | 6684 | 6648 |
| MDB0038 | 1 | Bogue Chitto | *A. plicata* | Mussel | 4548 | 3205 | 3512 | 3491 |
| MDB0039 | 1 | Bogue Chitto | *A. plicata* | Mussel | 6756 | 5231 | 5291 | 5269 |
| MDB0040 | 1 | Bogue Chitto | *A. plicata* | Mussel | 11046 | 8025 | 8252 | 8237 |
| MDB0042 | 1 | Bogue Chitto | *A. plicata* | Mussel | 6882 | 4964 | 5612 | 5591 |
| MDB0043 | 1 | Bogue Chitto | *A. plicata* | Mussel | 12028 | 7535 | 7901 | 7824 |
| MDB0044 | 1 | Bogue Chitto | *A. plicata* | Mussel | 8909 | 6284 | 7294 | 7274 |
| MDB0048 | 1 | Bogue Chitto | *L. ornata* | Mussel | 11022 | 7089 | 8348 | 8317 |
| MDB0051 | 2 | Bogue Chitto | *C. asperata* | Mussel | 5543 | 3608 | 4082 | 4065 |
| MDB0052 | 2 | Bogue Chitto | *C. asperata* | Mussel | 20762 | 14098 | 16622 | 16591 |
| MDB0054 | 2 | Bogue Chitto | *C. asperata* | Mussel | 6042 | 4139 | 4160 | 4151 |
| MDB0055 | 2 | Bogue Chitto | *C. asperata* | Mussel | 51163 | 37273 | 41874 | 41816 |
| MDB0056 | 2 | Bogue Chitto | *C. asperata* | Mussel | 74275 | 47886 | 56114 | 55912 |
| MDB0057 | 2 | Bogue Chitto | *C. asperata* | Mussel | 46416 | 26375 | 29526 | 29366 |
| MDB0064 | 2 | Bogue Chitto | *L. ornata* | Mussel | 6037 | 4300 | 5375 | 5344 |
| MDB0107 | 2 | Bear Creek | *A. plicata* | Mussel | 8659 | 3842 | 5434 | 5365 |
| MDB0108 | 2 | Bear Creek | *A. plicata* | Mussel | 30262 | 21052 | 23256 | 23201 |
| MDB0110 | 2 | Bear Creek | *A. plicata* | Mussel | 32441 | 18012 | 22500 | 22273 |
| MDB0111 | 2 | Bear Creek | *A. plicata* | Mussel | 17421 | 12470 | 13020 | 12996 |
| MDB0123 | 1 | Sipsey | *C. asperata* | Mussel | 21530 | 14097 | 14670 | 14604 |
| MDB0124 | 1 | Sipsey | *C. asperata* | Mussel | 12641 | 10355 | 8871 | 8854 |
| MDB0125 | 1 | Sipsey | *C. asperata* | Mussel | 21405 | 15959 | 14926 | 14894 |
| MDB0128 | 1 | Sipsey | *L. ornata* | Mussel | 6432 | 4634 | 3893 | 3879 |
| MDB0129 | 1 | Sipsey | *L. ornata* | Mussel | 21438 | 14287 | 16295 | 16038 |
| MDB0131 | 1 | Sipsey | *L. ornata* | Mussel | 43119 | 33750 | 35415 | 35262 |
| MDB0133 | 1 | Sipsey | *L. ornata* | Mussel | 9922 | 8362 | 5679 | 5657 |
| MDB0148 | 2 | Paint Rock | *A. plicata* | Mussel | 8087 | 6065 | 6657 | 6580 |
| MDB0201 | 1 | Buttahatchee | *C. asperata* | Mussel | 52850 | 31242 | 26128 | 26079 |
| MDB0202 | 1 | Buttahatchee | *C. asperata* | Mussel | 10274 | 6335 | 6573 | 6537 |
| MDB0203 | 1 | Buttahatchee | *C. asperata* | Mussel | 14847 | 10184 | 10985 | 10887 |
| MDB0209 | 1 | Buttahatchee | *C. asperata* | Mussel | 84549 | 62410 | 48821 | 48751 |
| MDB0220 | 1 | Buttahatchee | *L. ornata* | Mussel | 198354 | 112811 | 99952 | 98990 |
| MDB0221 | 1 | Buttahatchee | *L. ornata* | Mussel | 105971 | 67526 | 60439 | 60242 |
| MDB0223 | 1 | Buttahatchee | *L. ornata* | Mussel | 122993 | 83660 | 73112 | 72919 |
| MDB0225 | 1 | Buttahatchee | *L. ornata* | Mussel | 12774 | 8700 | 9840 | 9788 |
| MDB0226 | 1 | Buttahatchee | *L. ornata* | Mussel | 8624 | 5683 | 5655 | 5645 |
| MDB0229 | 1 | Buttahatchee | *L. ornata* | Mussel | 22532 | 18030 | 17351 | 17311 |
| MDB0230 | 2 | Buttahatchee | *C. asperata* | Mussel | 6144 | 4049 | 3486 | 3477 |
| MDB0231 | 2 | Buttahatchee | *C. asperata* | Mussel | 66577 | 32567 | 29099 | 29022 |
| MDB0232 | 2 | Buttahatchee | *C. asperata* | Mussel | 87437 | 65671 | 61434 | 61078 |
| MDB0233 | 2 | Buttahatchee | *C. asperata* | Mussel | 19588 | 14639 | 14529 | 14497 |
| MDB0234 | 2 | Buttahatchee | *C. asperata* | Mussel | 33525 | 16559 | 16945 | 16674 |
| MDB0235 | 2 | Buttahatchee | *C. asperata* | Mussel | 30260 | 16612 | 15870 | 15789 |
| MDB0236 | 2 | Buttahatchee | *C. asperata* | Mussel | 10995 | 8130 | 6884 | 6875 |
| MDB0237 | 2 | Buttahatchee | *C. asperata* | Mussel | 10198 | 6532 | 7054 | 6997 |
| MDB0238 | 2 | Buttahatchee | *C. asperata* | Mussel | 18527 | 11903 | 12756 | 12715 |
| MDB0239 | 2 | Buttahatchee | *C. asperata* | Mussel | 64667 | 42718 | 38875 | 38805 |
| MDB0270 | 2 | Buttahatchee | *L. ornata* | Mussel | 24231 | 16487 | 17697 | 17561 |
| MDB0272 | 2 | Buttahatchee | *L. ornata* | Mussel | 28748 | 6333 | 25260 | 25236 |
| MDB0276 | 2 | Buttahatchee | *L. ornata* | Mussel | 6468 | 3585 | 3666 | 3623 |
| MDB0279 | 2 | Buttahatchee | *L. ornata* | Mussel | 4513 | 3857 | 3437 | 3431 |
| MDB0282 | 2 | Sipsey | *C. asperata* | Mussel | 6951 | 4976 | 5754 | 5722 |
| MDB0284 | 2 | Sipsey | *C. asperata* | Mussel | 9797 | 6798 | 7713 | 7675 |
| MDB0285 | 2 | Sipsey | *C. asperata* | Mussel | 8704 | 6753 | 7471 | 7415 |
| MDB0288 | 2 | Sipsey | *C. asperata* | Mussel | 5064 | 3603 | 3611 | 3598 |
| MDB0310 | 2 | Sipsey | *L. ornata* | Mussel | 10846 | 6407 | 8268 | 8193 |
| MDB0311 | 2 | Sipsey | *L. ornata* | Mussel | 22686 | 15224 | 16201 | 16108 |
| MDB0313 | 2 | Sipsey | *L. ornata* | Mussel | 14479 | 8815 | 10458 | 10358 |
| MDB0314 | 2 | Sipsey | *L. ornata* | Mussel | 17236 | 8715 | 14048 | 14035 |
| MDB0315 | 2 | Sipsey | *L. ornata* | Mussel | 12826 | 7790 | 9457 | 9362 |
| MDB0316 | 2 | Sipsey | *L. ornata* | Mussel | 25087 | 7533 | 21321 | 21294 |
| MDB0317 | 2 | Sipsey | *L. ornata* | Mussel | 72822 | 53475 | 62334 | 62265 |
| MDB0318 | 2 | Sipsey | *L. ornata* | Mussel | 16220 | 3286 | 14521 | 14500 |
| MDB0324 | 3 | Sipsey | *C. asperata* | Mussel | 6038 | 5291 | 5232 | 5222 |
| MDB0325 | 3 | Sipsey | *C. asperata* | Mussel | 5231 | 4159 | 4255 | 4249 |
| MDB0326 | 3 | Sipsey | *C. asperata* | Mussel | 22291 | 18778 | 16845 | 16743 |
| MDB0331 | 3 | Sipsey | *C. asperata* | Mussel | 56695 | 39218 | 38812 | 38759 |
| MDB0332 | 3 | Sipsey | *C. asperata* | Mussel | 5449 | 3218 | 3864 | 3825 |
| MDB0375 | 3 | Sipsey | *A. plicata* | Mussel | 22071 | 17437 | 17640 | 17593 |
| MDB0377 | 3 | Sipsey | *L. ornata* | Mussel | 55529 | 30091 | 33171 | 32711 |
| MDB0378 | 3 | Sipsey | *L. ornata* | Mussel | 53059 | 27427 | 36907 | 36434 |
| MDB0379 | 3 | Sipsey | *L. ornata* | Mussel | 167221 | 104046 | 143827 | 143714 |
| MDB0381 | 3 | Sipsey | *L. ornata* | Mussel | 53958 | 42040 | 44126 | 43741 |
| MDB0382 | 3 | Sipsey | *L. ornata* | Mussel | 60609 | 40103 | 51663 | 51612 |
| MDB0385 | 3 | Sipsey | *L. ornata* | Mussel | 43360 | 21322 | 24533 | 24252 |
| MDB0386 | 4 | Sipsey | *C. asperata* | Mussel | 7752 | 4746 | 5291 | 5217 |
| MDB0387 | 4 | Sipsey | *C. asperata* | Mussel | 23368 | 18144 | 15454 | 15417 |
| MDB0388 | 4 | Sipsey | *C. asperata* | Mussel | 31997 | 19414 | 20141 | 20021 |
| MDB0390 | 4 | Sipsey | *C. asperata* | Mussel | 5069 | 3556 | 4152 | 4095 |
| MDB0394 | 4 | Sipsey | *C. asperata* | Mussel | 6714 | 4986 | 5498 | 5468 |
| MDB0395 | 4 | Sipsey | *C. asperata* | Mussel | 132638 | 104896 | 107613 | 107532 |
| MDB0457 | 4 | Sipsey | *L. ornata* | Mussel | 16306 | 4324 | 15154 | 15133 |
| MDB0461 | 4 | Sipsey | *L. ornata* | Mussel | 8889 | 6398 | 7370 | 7290 |
| MDB0462 | 4 | Sipsey | *L. ornata* | Mussel | 5036 | 4057 | 4376 | 4346 |
| MDB0503 | 1 | Paint Rock | *A. plicata* | Mussel | 7070 | 5677 | 5974 | 5953 |
| MDB0508 | 1 | Paint Rock | *A. plicata* | Mussel | 17128 | 21942 | 12300 | 12069 |
| MDB0561 | 4 | Duck | *A. plicata* | Mussel | 44637 | 37093 | 36745 | 36665 |
| MDB0562 | 4 | Duck | *A. plicata* | Mussel | 12801 | 7089 | 7233 | 7157 |
| MDB0566 | 4 | Duck | *A. plicata* | Mussel | 10510 | 9184 | 9297 | 9279 |
| MDB0567 | 4 | Duck | *A. plicata* | Mussel | 9091 | 6406 | 6416 | 6375 |
| MDB0569 | 5 | Sipsey | *C. asperata* | Mussel | 23816 | 5723 | 21959 | 21941 |
| MDB0570 | 5 | Sipsey | *C. asperata* | Mussel | 6460 | 4250 | 4186 | 4177 |
| MDB0571 | 5 | Sipsey | *C. asperata* | Mussel | 7650 | 12244 | 5761 | 5749 |
| MDB0573 | 5 | Sipsey | *C. asperata* | Mussel | 11652 | 7161 | 6651 | 6624 |
| MDB0574 | 5 | Sipsey | *C. asperata* | Mussel | 4877 | 4177 | 4140 | 4136 |
| MDB0575 | 5 | Sipsey | *C. asperata* | Mussel | 12648 | 8519 | 8571 | 8553 |
| MDB0577 | 5 | Sipsey | *C. asperata* | Mussel | 11344 | 8264 | 8052 | 8029 |
| MDB0614 | 5 | Sipsey | *A. plicata* | Mussel | 39746 | 29721 | 32513 | 32392 |
| MDB0615 | 5 | Sipsey | *A. plicata* | Mussel | 52840 | 36408 | 36049 | 35940 |
| MDB0622 | 5 | Sipsey | *A. plicata* | Mussel | 20855 | 13356 | 14499 | 14456 |
| MDB0623 | 5 | Sipsey | *L. ornata* | Mussel | 71831 | 39533 | 34838 | 34506 |
| MDB0624 | 5 | Sipsey | *L. ornata* | Mussel | 25987 | 12617 | 11729 | 11652 |
| MDB0625 | 5 | Sipsey | *L. ornata* | Mussel | 5740 | 4174 | 4583 | 4577 |
| MDB0626 | 5 | Sipsey | *L. ornata* | Mussel | 33797 | 17582 | 14570 | 14419 |
| MDB0627 | 5 | Sipsey | *L. ornata* | Mussel | 7657 | 4181 | 3910 | 3864 |
| MDB0628 | 5 | Sipsey | *L. ornata* | Mussel | 7216 | 4907 | 4698 | 4672 |
| MDB0629 | 5 | Sipsey | *L. ornata* | Mussel | 25172 | 8191 | 19445 | 19391 |
| MDB0630 | 5 | Sipsey | *L. ornata* | Mussel | 54317 | 29308 | 25322 | 24973 |
| MDB0631 | 5 | Sipsey | *L. ornata* | Mussel | 23863 | 13434 | 12872 | 12812 |
| MDB0632 | 5 | Sipsey | *L. ornata* | Mussel | 10582 | 7865 | 7695 | 7681 |
| **Mean Mussel Microbiome** | | | | | **27108±31945** | **17674±20812** | **18665±21676** | **18580±21612** |
| MDS0001 | 2 | Bear Creek | - | Sediment | 15241 | 8210 | 11356 | 11260 |
| MDS0002 | 2 | Bear Creek | - | Sediment | 20301 | 9971 | 15973 | 15611 |
| MDS0005 | 2 | Duck | - | Sediment | 9867 | 4042 | 7632 | 7330 |
| MDS0006 | 2 | Duck | - | Sediment | 16647 | 9821 | 13183 | 13128 |
| MDS0007 | 1 | Duck | - | Sediment | 27389 | 14087 | 21121 | 20515 |
| MDS0008 | 1 | Duck | - | Sediment | 14345 | 7357 | 11163 | 10790 |
| MDS0009 | 1 | Duck | - | Sediment | 14126 | 7847 | 10642 | 10491 |
| MDS0010 | 3 | Duck | - | Sediment | 31450 | 16587 | 24181 | 23205 |
| MDS0011 | 3 | Duck | - | Sediment | 14004 | 5411 | 10735 | 10063 |
| MDS0012 | 3 | Duck | - | Sediment | 16614 | 8033 | 12967 | 12524 |
| MDS0013 | 1 | Paint Rock | - | Sediment | 11866 | 5003 | 9027 | 8674 |
| MDS0014 | 1 | Paint Rock | - | Sediment | 8887 | 3939 | 6937 | 6725 |
| MDS0016 | 1 | Sipsey | - | Sediment | 15162 | 7791 | 12384 | 12064 |
| MDS0017 | 1 | Sipsey | - | Sediment | 10480 | 5081 | 7853 | 7776 |
| MDS0018 | 1 | Sipsey | - | Sediment | 15467 | 7279 | 12182 | 11967 |
| MDS0019 | 2 | Buttahatchee | - | Sediment | 14946 | 7376 | 11451 | 11348 |
| MDS0020 | 2 | Buttahatchee | - | Sediment | 25986 | 13141 | 19401 | 18991 |
| MDS0021 | 2 | Buttahatchee | - | Sediment | 8455 | 3889 | 6426 | 6324 |
| MDS0022 | 1 | Bogue Chitto | - | Sediment | 13335 | 4935 | 10425 | 9924 |
| MDS0023 | 1 | Bogue Chitto | - | Sediment | 14346 | 6194 | 10803 | 10279 |
| MDS0025 | 4 | Sipsey | - | Sediment | 19961 | 9964 | 13888 | 13803 |
| MDS0026 | 4 | Sipsey | - | Sediment | 22294 | 10814 | 15459 | 15313 |
| MDS0027 | 4 | Sipsey | - | Sediment | 14930 | 8461 | 11622 | 11478 |
| MDS0028 | 2 | Paint Rock | - | Sediment | 17166 | 8081 | 13294 | 12944 |
| MDS0033 | 1 | Buttahatchee | - | Sediment | 13564 | 8897 | 10573 | 10556 |
| MDS0034 | 2 | Bogue Chitto | - | Sediment | 9251 | 4420 | 6545 | 6449 |
| MDS0035 | 2 | Bogue Chitto | - | Sediment | 17429 | 8964 | 12720 | 12481 |
| MDS0036 | 2 | Bogue Chitto | - | Sediment | 12264 | 6177 | 8846 | 8688 |
| MDS0038 | 3 | Paint Rock | - | Sediment | 9040 | 4506 | 7044 | 6908 |
| MDS0039 | 3 | Paint Rock | - | Sediment | 8022 | 3742 | 6144 | 5964 |
| MDS0040 | 1 | Bear Creek | - | Sediment | 8555 | 3369 | 6088 | 5970 |
| MDS0041 | 1 | Bear Creek | - | Sediment | 21227 | 12470 | 15106 | 14860 |
| MDS0042 | 1 | Bear Creek | - | Sediment | 16174 | 11148 | 10521 | 10480 |
| MDS0043 | 5 | Sipsey | - | Sediment | 20879 | 12921 | 14807 | 14679 |
| MDS0044 | 5 | Sipsey | - | Sediment | 17115 | 8737 | 13048 | 12876 |
| MDS0045 | 5 | Sipsey | - | Sediment | 8273 | 3423 | 6399 | 6230 |
| MDS0046 | 4 | Duck | - | Sediment | 13531 | 6878 | 11119 | 10919 |
| MDS0047 | 4 | Duck | - | Sediment | 9403 | 5134 | 7447 | 7339 |
| MDS0048 | 4 | Duck | - | Sediment | 15118 | 8670 | 11276 | 11138 |
| MDS0049 | 2 | Sipsey | - | Sediment | 12489 | 5509 | 10039 | 9671 |
| MDS0050 | 2 | Sipsey | - | Sediment | 9337 | 4405 | 7286 | 6980 |
| MDS0051 | 2 | Sipsey | - | Sediment | 13745 | 6439 | 10956 | 10502 |
| MDS0052 | 3 | Sipsey | - | Sediment | 24032 | 13232 | 19186 | 18803 |
| MDS0053 | 3 | Sipsey | - | Sediment | 20694 | 12473 | 15991 | 15737 |
| MDS0054 | 3 | Sipsey | - | Sediment | 11309 | 4779 | 8877 | 8637 |
| **Mean Sediment** | | | | | **15216±5437** | **7769±3289** | **11558±4097** | **11298±4000** |
| MDW0001 | 2 | Bear Creek | - | Seston | 34949 | 17952 | 22409 | 21944 |
| MDW0002 | 2 | Bear Creek | - | Seston | 30196 | 15112 | 19129 | 18728 |
| MDW0003 | 2 | Bear Creek | - | Seston | 19154 | 9877 | 12559 | 12304 |
| MDW0004 | 2 | Duck | - | Seston | 15623 | 7790 | 10584 | 10425 |
| MDW0005 | 2 | Duck | - | Seston | 26940 | 13658 | 17820 | 17568 |
| MDW0006 | 2 | Duck | - | Seston | 37976 | 18652 | 24464 | 24099 |
| MDW0007 | 1 | Duck | - | Seston | 23108 | 11159 | 15119 | 14894 |
| MDW0008 | 1 | Duck | - | Seston | 19298 | 9362 | 12771 | 12577 |
| MDW0009 | 1 | Duck | - | Seston | 35010 | 18393 | 23487 | 23141 |
| MDW0010 | 3 | Duck | - | Seston | 29014 | 15403 | 18963 | 18712 |
| MDW0011 | 3 | Duck | - | Seston | 23594 | 13591 | 16390 | 16181 |
| MDW0012 | 3 | Duck | - | Seston | 22172 | 11672 | 14713 | 14492 |
| MDW0013 | 1 | Paint Rock | - | Seston | 27367 | 10971 | 13977 | 13743 |
| MDW0014 | 1 | Paint Rock | - | Seston | 50141 | 23030 | 27487 | 27054 |
| MDW0015 | 1 | Paint Rock | - | Seston | 26881 | 11171 | 13971 | 13730 |
| MDW0016 | 1 | Sipsey | - | Seston | 29080 | 16317 | 20269 | 20024 |
| MDW0017 | 1 | Sipsey | - | Seston | 57113 | 34963 | 38893 | 38480 |
| MDW0018 | 1 | Sipsey | - | Seston | 23041 | 12119 | 15030 | 14649 |
| MDW0019 | 2 | Buttahatchee | - | Seston | 21403 | 11443 | 13352 | 13117 |
| MDW0020 | 2 | Buttahatchee | - | Seston | 19170 | 8975 | 11162 | 10883 |
| MDW0021 | 2 | Buttahatchee | - | Seston | 27368 | 13722 | 16403 | 16077 |
| MDW0022 | 1 | Bogue Chitto | - | Seston | 8923 | 3886 | 6107 | 5984 |
| MDW0023 | 1 | Bogue Chitto | - | Seston | 14286 | 6132 | 9193 | 8944 |
| MDW0024 | 1 | Bogue Chitto | - | Seston | 8645 | 3608 | 5973 | 5775 |
| MDW0025 | 4 | Sipsey | - | Seston | 15006 | 8104 | 10420 | 10265 |
| MDW0026 | 4 | Sipsey | - | Seston | 10266 | 5745 | 7325 | 7218 |
| MDW0027 | 4 | Sipsey | - | Seston | 10438 | 5552 | 7252 | 7135 |
| MDW0028 | 2 | Paint Rock | - | Seston | 11951 | 4736 | 6418 | 6240 |
| MDW0029 | 2 | Paint Rock | - | Seston | 8498 | 3252 | 4469 | 4340 |
| MDW0031 | 1 | Buttahatchee | - | Seston | 8133 | 4350 | 5264 | 5140 |
| MDW0032 | 1 | Buttahatchee | - | Seston | 9239 | 5294 | 6083 | 5972 |
| MDW0033 | 1 | Buttahatchee | - | Seston | 8455 | 3983 | 5077 | 4964 |
| MDW0034 | 2 | Bogue Chitto | - | Seston | 10552 | 3972 | 6782 | 6580 |
| MDW0035 | 2 | Bogue Chitto | - | Seston | 10595 | 3957 | 6799 | 6570 |
| MDW0036 | 2 | Bogue Chitto | - | Seston | 8595 | 3557 | 5394 | 5267 |
| MDW0037 | 3 | Paint Rock | - | Seston | 10371 | 4259 | 5929 | 5744 |
| MDW0038 | 3 | Paint Rock | - | Seston | 10475 | 4590 | 6133 | 5947 |
| MDW0039 | 3 | Paint Rock | - | Seston | 10761 | 4741 | 6397 | 6204 |
| MDW0040 | 1 | Bear Creek | - | Seston | 7493 | 3603 | 4973 | 4830 |
| MDW0041 | 1 | Bear Creek | - | Seston | 9501 | 5027 | 6308 | 6175 |
| MDW0042 | 1 | Bear Creek | - | Seston | 7947 | 3765 | 5321 | 5182 |
| MDW0043 | 5 | Sipsey | - | Seston | 9541 | 5272 | 6531 | 6428 |
| MDW0045 | 5 | Sipsey | - | Seston | 11767 | 6130 | 7851 | 7704 |
| MDW0046 | 4 | Duck | - | Seston | 9034 | 5049 | 5768 | 5704 |
| MDW0047 | 4 | Duck | - | Seston | 9287 | 5425 | 6199 | 6124 |
| MDW0048 | 4 | Duck | - | Seston | 7958 | 4808 | 5312 | 5252 |
| MDW0049 | 4 | Sipsey | - | Seston | 7984 | 4249 | 5627 | 5522 |
| MDW0051 | 2 | Sipsey | - | Seston | 8264 | 3643 | 5134 | 5056 |
| MDW0052 | 2 | Sipsey | - | Seston | 10313 | 4639 | 6714 | 6550 |
| MDW0053 | 3 | Sipsey | - | Seston | 18406 | 7422 | 10613 | 10352 |
| MDW0055 | 3 | Sipsey | - | Seston | 12257 | 4831 | 7126 | 6969 |
| **Mean Seston** | | | | | **17717±11305** | **8802±6222** | **11244±7189** | **11038±7105** |

**S3 Table:** Sequencing statistics for each sample of study, on forward and reverse reads assessed by fastqc. pct.dup: percentage of duplicate reads ; pct.gc: percentage of GC content; seq.length: range of sequence length; status: fastqc module status for per sequence average quality score.

|  | **Forward reads** | | | | **Reverse reads** | | | |
| --- | --- | --- | --- | --- | --- | --- | --- | --- |
| **Sample** | **pct.dup** | **pct.gc** | **seq.length** | **status** | **pct.dup** | **pct.gc** | **seq.length** | **status** |
| MDB0015 | 94.74 | 51 | 248-251 | PASS | 89.72 | 52 | 247-251 | PASS |
| MDB0016 | 92.44 | 53 | 248-251 | PASS | 87.69 | 53 | 246-251 | PASS |
| MDB0017 | 93.8 | 52 | 247-251 | PASS | 88.95 | 52 | 247-251 | PASS |
| MDB0018 | 94.73 | 53 | 249-251 | PASS | 91.11 | 54 | 246-251 | PASS |
| MDB0019 | 94.19 | 53 | 248-251 | PASS | 91 | 53 | 247-251 | PASS |
| MDB0021 | 92.02 | 52 | 248-251 | PASS | 87.9 | 53 | 246-251 | PASS |
| MDB0023 | 95.22 | 52 | 249-251 | PASS | 90.3 | 53 | 245-251 | PASS |
| MDB0024 | 95.74 | 52 | 247-251 | PASS | 92.68 | 52 | 247-251 | PASS |
| MDB0036 | 93.4 | 52 | 248-251 | PASS | 87.19 | 53 | 245-251 | PASS |
| MDB0037 | 90.6 | 54 | 248-251 | PASS | 83.32 | 54 | 247-251 | PASS |
| MDB0038 | 88.94 | 53 | 249-251 | PASS | 83.05 | 54 | 247-251 | PASS |
| MDB0039 | 92.7 | 54 | 244-251 | PASS | 87.49 | 54 | 247-251 | PASS |
| MDB0040 | 93.1 | 54 | 249-251 | PASS | 82.62 | 55 | 247-251 | PASS |
| MDB0042 | 92.24 | 53 | 248-251 | PASS | 86.78 | 54 | 247-251 | PASS |
| MDB0043 | 90.97 | 53 | 248-251 | PASS | 83.95 | 53 | 245-251 | PASS |
| MDB0044 | 92.89 | 53 | 249-251 | PASS | 87.36 | 53 | 246-251 | PASS |
| MDB0048 | 92.85 | 53 | 248-251 | PASS | 82.88 | 53 | 247-251 | PASS |
| MDB0051 | 89.59 | 53 | 249-251 | PASS | 82.46 | 53 | 246-251 | PASS |
| MDB0052 | 94.6 | 52 | 249-251 | PASS | 90.06 | 53 | 246-251 | PASS |
| MDB0054 | 91.69 | 52 | 247-251 | PASS | 86.4 | 53 | 247-251 | PASS |
| MDB0055 | 96.15 | 52 | 248-251 | PASS | 92.59 | 53 | 246-251 | PASS |
| MDB0056 | 95.79 | 53 | 248-251 | PASS | 86.99 | 53 | 246-251 | PASS |
| MDB0057 | 94.31 | 52 | 248-251 | PASS | 89.15 | 52 | 245-251 | PASS |
| MDB0064 | 91.85 | 53 | 248-251 | PASS | 86.96 | 54 | 246-251 | PASS |
| MDB0107 | 89.59 | 52 | 246-251 | PASS | 82.35 | 52 | 247-251 | PASS |
| MDB0108 | 95.45 | 51 | 247-251 | PASS | 89.82 | 52 | 247-251 | PASS |
| MDB0110 | 93.34 | 53 | 248-251 | PASS | 85.63 | 53 | 245-251 | PASS |
| MDB0111 | 94.88 | 53 | 247-251 | PASS | 88.97 | 54 | 247-251 | PASS |
| MDB0123 | 92.6 | 53 | 248-251 | PASS | 80.85 | 53 | 247-251 | PASS |
| MDB0124 | 93.94 | 51 | 247-251 | PASS | 83.63 | 52 | 247-251 | PASS |
| MDB0125 | 94.14 | 52 | 248-251 | PASS | 81.93 | 53 | 245-251 | PASS |
| MDB0128 | 91.88 | 52 | 248-251 | PASS | 68.08 | 53 | 246-251 | PASS |
| MDB0129 | 91.51 | 53 | 249-251 | PASS | 77.49 | 54 | 247-251 | PASS |
| MDB0131 | 94.75 | 54 | 248-251 | PASS | 89.36 | 55 | 246-251 | PASS |
| MDB0133 | 93.55 | 53 | 247-251 | PASS | 84.97 | 53 | 246-251 | PASS |
| MDB0148 | 90.49 | 55 | 248-251 | PASS | 84.78 | 55 | 247-251 | PASS |
| MDB0201 | 95.8 | 51 | 247-251 | PASS | 89.99 | 52 | 246-251 | PASS |
| MDB0202 | 90.85 | 52 | 248-251 | PASS | 82.4 | 52 | 247-251 | PASS |
| MDB0203 | 92.17 | 53 | 247-251 | PASS | 84.78 | 53 | 247-251 | PASS |
| MDB0209 | 96.58 | 52 | 247-251 | PASS | 88.21 | 52 | 244-251 | PASS |
| MDB0220 | 95.84 | 52 | 248-251 | PASS | 87.32 | 53 | 245-251 | PASS |
| MDB0221 | 96 | 51 | 247-251 | PASS | 82.61 | 51 | 245-251 | PASS |
| MDB0223 | 96.83 | 51 | 247-251 | PASS | 86.73 | 52 | 245-251 | PASS |
| MDB0225 | 91.91 | 52 | 248-251 | PASS | 84.62 | 53 | 246-251 | PASS |
| MDB0226 | 91.66 | 51 | 247-251 | PASS | 85.18 | 52 | 245-251 | PASS |
| MDB0229 | 95.28 | 53 | 248-251 | PASS | 89.94 | 53 | 246-251 | PASS |
| MDB0230 | 91.05 | 52 | 248-251 | PASS | 82.28 | 53 | 247-251 | PASS |
| MDB0231 | 96.07 | 50 | 248-251 | PASS | 91.34 | 51 | 246-251 | PASS |
| MDB0232 | 95.39 | 53 | 248-251 | PASS | 89.27 | 54 | 245-251 | PASS |
| MDB0233 | 93.78 | 53 | 249-251 | PASS | 86.01 | 54 | 247-251 | PASS |
| MDB0234 | 92.32 | 52 | 248-251 | PASS | 84.67 | 52 | 246-251 | PASS |
| MDB0235 | 93.98 | 51 | 248-251 | PASS | 88.19 | 51 | 245-251 | PASS |
| MDB0236 | 93.04 | 53 | 247-251 | PASS | 80.95 | 54 | 246-251 | PASS |
| MDB0237 | 89.8 | 52 | 248-251 | PASS | 80.08 | 53 | 246-251 | PASS |
| MDB0238 | 92.62 | 54 | 248-251 | PASS | 80.71 | 55 | 247-251 | PASS |
| MDB0239 | 95.73 | 53 | 247-251 | PASS | 89.23 | 53 | 245-251 | PASS |
| MDB0270 | 94.13 | 51 | 248-251 | PASS | 89.27 | 52 | 246-251 | PASS |
| MDB0272 | 98 | 52 | 248-251 | PASS | 95.15 | 53 | 247-251 | PASS |
| MDB0276 | 90.03 | 51 | 248-251 | PASS | 84.4 | 52 | 246-251 | PASS |
| MDB0279 | 94.33 | 52 | 249-251 | PASS | 88.26 | 53 | 247-251 | PASS |
| MDB0282 | 90.53 | 53 | 248-251 | PASS | 85.07 | 54 | 247-251 | PASS |
| MDB0284 | 92.49 | 53 | 249-251 | PASS | 84.57 | 54 | 247-251 | PASS |
| MDB0285 | 91.5 | 54 | 246-251 | PASS | 85.79 | 55 | 246-251 | PASS |
| MDB0288 | 91.79 | 52 | 248-251 | PASS | 80.67 | 52 | 247-251 | PASS |
| MDB0310 | 91 | 53 | 248-251 | PASS | 84.18 | 53 | 246-251 | PASS |
| MDB0311 | 92.44 | 53 | 248-251 | PASS | 86.18 | 53 | 246-251 | PASS |
| MDB0313 | 91.19 | 53 | 248-251 | PASS | 82.76 | 53 | 247-251 | PASS |
| MDB0314 | 95.31 | 52 | 248-251 | PASS | 91.2 | 53 | 247-251 | PASS |
| MDB0315 | 90.96 | 53 | 248-251 | PASS | 83.07 | 54 | 246-251 | PASS |
| MDB0316 | 96.54 | 52 | 248-251 | PASS | 92.61 | 53 | 247-251 | PASS |
| MDB0317 | 97.3 | 52 | 247-251 | PASS | 94.79 | 53 | 247-251 | PASS |
| MDB0318 | 96.55 | 52 | 249-251 | PASS | 94.02 | 53 | 246-251 | PASS |
| MDB0324 | 93.28 | 51 | 249-251 | PASS | 89.58 | 52 | 247-251 | PASS |
| MDB0325 | 93.16 | 50 | 248-251 | PASS | 87.77 | 51 | 247-251 | PASS |
| MDB0326 | 94.78 | 56 | 249-251 | PASS | 88.77 | 57 | 247-251 | PASS |
| MDB0331 | 96.08 | 52 | 247-251 | PASS | 90.93 | 52 | 247-251 | PASS |
| MDB0332 | 87.8 | 53 | 248-251 | PASS | 76.99 | 54 | 247-251 | PASS |
| MDB0375 | 95.07 | 52 | 246-251 | PASS | 90.71 | 53 | 247-251 | PASS |
| MDB0377 | 94 | 51 | 248-251 | PASS | 82.12 | 52 | 246-251 | PASS |
| MDB0378 | 94.19 | 53 | 248-251 | PASS | 87.76 | 53 | 246-251 | PASS |
| MDB0379 | 98.51 | 52 | 247-251 | PASS | 95.31 | 53 | 246-251 | PASS |
| MDB0381 | 95.53 | 55 | 248-251 | PASS | 90.48 | 56 | 246-251 | PASS |
| MDB0382 | 98.22 | 55 | 248-251 | PASS | 95.09 | 56 | 246-251 | PASS |
| MDB0385 | 93.6 | 52 | 248-251 | PASS | 80.22 | 53 | 245-251 | PASS |
| MDB0386 | 87.65 | 52 | 248-251 | PASS | 77.72 | 52 | 247-251 | PASS |
| MDB0387 | 92.58 | 53 | 248-251 | PASS | 82.12 | 53 | 245-251 | PASS |
| MDB0388 | 93.96 | 51 | 248-251 | PASS | 88.24 | 52 | 247-251 | PASS |
| MDB0390 | 88.03 | 53 | 248-251 | PASS | 79.25 | 53 | 247-251 | PASS |
| MDB0394 | 90.69 | 52 | 248-251 | PASS | 82.28 | 53 | 247-251 | PASS |
| MDB0395 | 97.64 | 50 | 246-251 | PASS | 94.33 | 51 | 247-251 | PASS |
| MDB0457 | 96.73 | 52 | 248-251 | PASS | 93.93 | 53 | 247-251 | PASS |
| MDB0461 | 90.22 | 53 | 249-251 | PASS | 82.97 | 54 | 245-251 | PASS |
| MDB0462 | 90.93 | 51 | 248-251 | PASS | 86.12 | 52 | 246-251 | PASS |
| MDB0503 | 92.59 | 52 | 247-251 | PASS | 88.54 | 53 | 247-251 | PASS |
| MDB0508 | 90.57 | 53 | 247-251 | PASS | 83.1 | 54 | 246-251 | PASS |
| MDB0561 | 97.11 | 53 | 248-251 | PASS | 94.33 | 53 | 245-251 | PASS |
| MDB0562 | 93.16 | 51 | 248-251 | PASS | 88.62 | 52 | 247-251 | PASS |
| MDB0566 | 95.26 | 53 | 248-251 | PASS | 92.04 | 53 | 246-251 | PASS |
| MDB0567 | 93.74 | 53 | 248-251 | PASS | 88.6 | 53 | 246-251 | PASS |
| MDB0569 | 97.58 | 52 | 248-251 | PASS | 94.7 | 52 | 247-251 | PASS |
| MDB0570 | 92.79 | 51 | 245-251 | PASS | 88.56 | 51 | 247-251 | PASS |
| MDB0571 | 92.48 | 52 | 249-251 | PASS | 87.19 | 53 | 247-251 | PASS |
| MDB0573 | 93.26 | 51 | 248-251 | PASS | 88.29 | 51 | 247-251 | PASS |
| MDB0574 | 94.03 | 50 | 247-251 | PASS | 89.69 | 51 | 247-251 | PASS |
| MDB0575 | 94.71 | 50 | 247-251 | PASS | 91.17 | 50 | 245-251 | PASS |
| MDB0577 | 94.53 | 51 | 248-251 | PASS | 89.49 | 51 | 246-251 | PASS |
| MDB0614 | 96.16 | 53 | 248-251 | PASS | 91.09 | 54 | 108-251 | PASS |
| MDB0615 | 96.99 | 51 | 248-251 | PASS | 93.43 | 52 | 246-251 | PASS |
| MDB0622 | 95.44 | 52 | 248-251 | PASS | 89.94 | 52 | 247-251 | PASS |
| MDB0623 | 95.6 | 51 | 248-251 | PASS | 92.18 | 52 | 246-251 | PASS |
| MDB0624 | 94.53 | 50 | 248-251 | PASS | 89.66 | 51 | 246-251 | PASS |
| MDB0625 | 94.23 | 51 | 248-251 | PASS | 88.28 | 52 | 247-251 | PASS |
| MDB0626 | 95.06 | 51 | 248-251 | PASS | 91.05 | 52 | 246-251 | PASS |
| MDB0627 | 91.79 | 52 | 249-251 | PASS | 86.08 | 52 | 246-251 | PASS |
| MDB0628 | 92.99 | 52 | 249-251 | PASS | 84.3 | 52 | 247-251 | PASS |
| MDB0629 | 96.31 | 52 | 248-251 | PASS | 92.4 | 52 | 246-251 | PASS |
| MDB0630 | 94.78 | 51 | 248-251 | PASS | 89.11 | 52 | 245-251 | PASS |
| MDB0631 | 95.28 | 51 | 248-251 | PASS | 90.63 | 51 | 246-251 | PASS |
| MDB0632 | 94.46 | 51 | 248-251 | PASS | 89.91 | 52 | 247-251 | PASS |
| MDS0001 | 89.52 | 55 | 248-251 | PASS | 80.35 | 55 | 247-251 | PASS |
| MDS0002 | 87.15 | 56 | 249-251 | PASS | 76.29 | 56 | 247-251 | PASS |
| MDS0005 | 78.39 | 56 | 249-251 | PASS | 65.05 | 56 | 247-251 | PASS |
| MDS0006 | 90.24 | 56 | 249-251 | PASS | 83.1 | 56 | 247-251 | PASS |
| MDS0007 | 87.39 | 56 | 248-251 | PASS | 75.05 | 57 | 247-251 | PASS |
| MDS0008 | 83.37 | 56 | 249-251 | PASS | 72.59 | 57 | 247-251 | PASS |
| MDS0009 | 87.42 | 56 | 249-251 | PASS | 76.24 | 57 | 246-251 | PASS |
| MDS0010 | 88.03 | 55 | 249-251 | PASS | 77.06 | 56 | 245-251 | PASS |
| MDS0011 | 78.61 | 55 | 248-251 | PASS | 63.83 | 56 | 247-251 | PASS |
| MDS0012 | 84.33 | 56 | 248-251 | PASS | 71.45 | 57 | 246-251 | PASS |
| MDS0013 | 82.11 | 55 | 248-251 | PASS | 68.64 | 56 | 245-251 | PASS |
| MDS0014 | 80.38 | 55 | 247-251 | PASS | 67.22 | 56 | 247-251 | PASS |
| MDS0016 | 84.36 | 56 | 248-251 | PASS | 72.8 | 56 | 247-251 | PASS |
| MDS0017 | 86.92 | 55 | 249-251 | PASS | 74.64 | 56 | 247-251 | PASS |
| MDS0018 | 85.87 | 55 | 248-251 | PASS | 73.41 | 56 | 247-251 | PASS |
| MDS0019 | 89.07 | 55 | 248-251 | PASS | 75.25 | 56 | 247-251 | PASS |
| MDS0020 | 89.4 | 55 | 249-251 | PASS | 75.89 | 56 | 246-251 | PASS |
| MDS0021 | 84.9 | 55 | 249-251 | PASS | 69.43 | 56 | 247-251 | PASS |
| MDS0022 | 81.08 | 55 | 248-251 | PASS | 67.02 | 56 | 247-251 | PASS |
| MDS0023 | 80.34 | 55 | 248-251 | PASS | 62.72 | 56 | 247-251 | PASS |
| MDS0025 | 90.54 | 54 | 248-251 | PASS | 75.48 | 55 | 247-251 | PASS |
| MDS0026 | 90.26 | 55 | 249-251 | PASS | 76.97 | 55 | 245-251 | PASS |
| MDS0027 | 87.87 | 55 | 248-251 | PASS | 79.32 | 56 | 246-251 | PASS |
| MDS0028 | 84.57 | 54 | 248-251 | PASS | 74.58 | 55 | 247-251 | PASS |
| MDS0033 | 93.25 | 55 | 249-251 | PASS | 88.04 | 55 | 246-251 | PASS |
| MDS0034 | 84.57 | 54 | 249-251 | PASS | 74.12 | 54 | 247-251 | PASS |
| MDS0035 | 86.8 | 54 | 249-251 | PASS | 77.4 | 54 | 247-251 | PASS |
| MDS0036 | 84.03 | 54 | 248-251 | PASS | 72.24 | 55 | 245-251 | PASS |
| MDS0038 | 84.13 | 54 | 249-251 | PASS | 72.58 | 55 | 247-251 | PASS |
| MDS0039 | 80.9 | 54 | 249-251 | PASS | 67.74 | 55 | 246-251 | PASS |
| MDS0040 | 81.78 | 54 | 248-251 | PASS | 66.35 | 55 | 247-251 | PASS |
| MDS0041 | 88.78 | 55 | 250-251 | PASS | 79.17 | 56 | 247-251 | PASS |
| MDS0042 | 92.85 | 55 | 248-251 | PASS | 84.75 | 55 | 247-251 | PASS |
| MDS0043 | 90.6 | 55 | 249-251 | PASS | 82.4 | 56 | 246-251 | PASS |
| MDS0044 | 87.04 | 55 | 248-251 | PASS | 77.94 | 56 | 247-251 | PASS |
| MDS0045 | 79.21 | 55 | 248-251 | PASS | 67.09 | 56 | 246-251 | PASS |
| MDS0046 | 85.27 | 56 | 249-251 | PASS | 77.06 | 57 | 247-251 | PASS |
| MDS0047 | 83.98 | 56 | 248-251 | PASS | 74.51 | 57 | 247-251 | PASS |
| MDS0048 | 87.95 | 56 | 248-251 | PASS | 78.56 | 57 | 246-251 | PASS |
| MDS0049 | 79.43 | 56 | 249-251 | PASS | 68.14 | 56 | 246-251 | PASS |
| MDS0050 | 76.17 | 55 | 249-251 | PASS | 65.31 | 56 | 247-251 | PASS |
| MDS0051 | 80.41 | 55 | 247-251 | PASS | 69.75 | 56 | 247-251 | PASS |
| MDS0052 | 88.05 | 56 | 247-251 | PASS | 79.77 | 57 | 244-251 | PASS |
| MDS0053 | 90.07 | 55 | 249-251 | PASS | 81.29 | 56 | 247-251 | PASS |
| MDS0054 | 81.06 | 55 | 249-251 | PASS | 69.34 | 55 | 247-251 | PASS |
| MDW0001 | 90.03 | 51 | 245-251 | PASS | 82.37 | 52 | 245-251 | PASS |
| MDW0002 | 88.72 | 52 | 248-251 | PASS | 78.15 | 52 | 245-251 | PASS |
| MDW0003 | 87.21 | 52 | 245-251 | PASS | 78.15 | 52 | 245-251 | PASS |
| MDW0004 | 88.11 | 52 | 247-251 | PASS | 79.38 | 52 | 245-251 | PASS |
| MDW0005 | 89.99 | 52 | 247-251 | PASS | 83.19 | 52 | 245-251 | PASS |
| MDW0006 | 91.17 | 52 | 248-251 | PASS | 81.65 | 52 | 245-251 | PASS |
| MDW0007 | 89.64 | 52 | 248-251 | PASS | 80.08 | 52 | 245-251 | PASS |
| MDW0008 | 88.84 | 52 | 247-251 | PASS | 79.53 | 52 | 246-251 | PASS |
| MDW0009 | 91.17 | 52 | 248-251 | PASS | 82.44 | 52 | 245-251 | PASS |
| MDW0010 | 90.22 | 51 | 248-251 | PASS | 76.75 | 52 | 245-251 | PASS |
| MDW0011 | 89.82 | 52 | 247-251 | PASS | 78.89 | 52 | 245-251 | PASS |
| MDW0012 | 89.46 | 52 | 248-251 | PASS | 76.81 | 52 | 245-251 | PASS |
| MDW0013 | 90.79 | 51 | 247-251 | PASS | 81.25 | 51 | 245-251 | PASS |
| MDW0014 | 92.44 | 51 | 247-251 | PASS | 83.75 | 52 | 245-251 | PASS |
| MDW0015 | 90.39 | 51 | 247-251 | PASS | 81.49 | 52 | 245-251 | PASS |
| MDW0016 | 90.41 | 52 | 248-251 | PASS | 80.42 | 52 | 245-251 | PASS |
| MDW0017 | 92.49 | 52 | 248-251 | PASS | 83.07 | 53 | 245-251 | PASS |
| MDW0018 | 86.45 | 53 | 248-251 | PASS | 70.86 | 54 | 245-251 | PASS |
| MDW0019 | 89.57 | 52 | 248-251 | PASS | 79.06 | 53 | 245-251 | PASS |
| MDW0020 | 88.71 | 52 | 248-251 | PASS | 75.76 | 53 | 246-251 | PASS |
| MDW0021 | 90.13 | 52 | 248-251 | PASS | 80.16 | 53 | 245-251 | PASS |
| MDW0022 | 83.55 | 52 | 248-251 | PASS | 73.85 | 53 | 245-251 | PASS |
| MDW0023 | 84.23 | 52 | 248-251 | PASS | 74.77 | 53 | 245-251 | PASS |
| MDW0024 | 79.83 | 53 | 248-251 | PASS | 66.64 | 53 | 245-251 | PASS |
| MDW0025 | 88.95 | 52 | 248-251 | PASS | 79.58 | 53 | 245-251 | PASS |
| MDW0026 | 87.41 | 52 | 248-251 | PASS | 78.93 | 53 | 245-251 | PASS |
| MDW0027 | 87.07 | 52 | 249-251 | PASS | 77.68 | 53 | 245-251 | PASS |
| MDW0028 | 85.88 | 51 | 247-251 | PASS | 77.7 | 51 | 245-251 | PASS |
| MDW0029 | 84.7 | 51 | 247-251 | PASS | 74.92 | 52 | 245-251 | PASS |
| MDW0031 | 85.07 | 52 | 248-251 | PASS | 76.37 | 53 | 246-251 | PASS |
| MDW0032 | 87.6 | 52 | 248-251 | PASS | 79.08 | 53 | 247-251 | PASS |
| MDW0033 | 85.7 | 52 | 248-251 | PASS | 73.47 | 52 | 246-251 | PASS |
| MDW0034 | 81.97 | 52 | 248-251 | PASS | 69.66 | 53 | 245-251 | PASS |
| MDW0035 | 82.12 | 52 | 248-251 | PASS | 68.32 | 53 | 245-251 | PASS |
| MDW0036 | 84.11 | 52 | 248-251 | PASS | 74.46 | 52 | 245-251 | PASS |
| MDW0037 | 84.22 | 51 | 247-251 | PASS | 75.8 | 52 | 245-251 | PASS |
| MDW0038 | 84.46 | 52 | 247-251 | PASS | 76.12 | 52 | 245-251 | PASS |
| MDW0039 | 84.49 | 52 | 248-251 | PASS | 76.29 | 52 | 245-251 | PASS |
| MDW0040 | 82.62 | 52 | 248-251 | PASS | 73.78 | 53 | 245-251 | PASS |
| MDW0041 | 84.7 | 52 | 248-251 | PASS | 74.28 | 53 | 245-251 | PASS |
| MDW0042 | 83.08 | 52 | 249-251 | PASS | 74.38 | 52 | 245-251 | PASS |
| MDW0043 | 86.35 | 53 | 248-251 | PASS | 77.26 | 53 | 245-251 | PASS |
| MDW0045 | 86.66 | 52 | 248-251 | PASS | 77.9 | 53 | 245-251 | PASS |
| MDW0046 | 89.22 | 52 | 248-251 | PASS | 83.33 | 52 | 245-251 | PASS |
| MDW0047 | 89.21 | 52 | 248-251 | PASS | 84.19 | 52 | 245-251 | PASS |
| MDW0048 | 88.89 | 52 | 248-251 | PASS | 83.43 | 52 | 245-251 | PASS |
| MDW0049 | 85.65 | 52 | 248-251 | PASS | 75.88 | 53 | 246-251 | PASS |
| MDW0051 | 85.87 | 51 | 248-251 | PASS | 74.94 | 52 | 245-251 | PASS |
| MDW0052 | 84.19 | 52 | 248-251 | PASS | 73.91 | 52 | 245-251 | PASS |
| MDW0053 | 87.14 | 51 | 248-251 | PASS | 78.84 | 52 | 245-251 | PASS |
| MDW0055 | 85.54 | 51 | 248-251 | PASS | 77.38 | 52 | 245-251 | PASS |

**S4 Table: Average (±Standard Deviation) of percentage of abundance of top 30 genera in ASVs, 97%-OTUs and 99%-OTUs datasets, after rarefaction to 2,000 sequences per sample**. For this comparison, biological units that remained unclassified at Family level (representing 10.8 %, 19.4 % and 19.2% of abundance, respectively within ASV, 97%-OTUs and 99%-OTUs datasets) were removed. Correlation of ranks of abundance of each genus between ASVs and OTUs datasets was tested using separated Spearman’s signed rank tests for each genus and for each OTU threshold. Genera that showed poor reproducibility across methods (Spearman’s r<0.9 in ASVs vs. OTUs comparison), or that remained undetected in a given method (‘0’), were highlighted in bold.

| **Family** | **Genus** | **ASVs** | **97%-OTUs** | **99%-OTUs** |
| --- | --- | --- | --- | --- |
| Gemmataceae | unclassified | 11.1±11.7% | 10.3±10.6% | 10.3±10.8% |
| Mycoplasmataceae | unclassified | 7.8±12.3% | 7±11.3% | 7±11.4% |
| Beijerinckiaceae | *Methylocystis* | 6.4±8.6% | **3.5±8%** | **3.4±8%** |
| Cyanobiaceae | *Cyanobium_PCC-6307* | 4.8±10.8% | **8.3±11.9%** | **8.4±12%** |
| Pirellulaceae | unclassified | 4.5±6.3% | 4.7±5.6% | 4.7±5.7% |
| Peptostreptococcaceae | *Romboutsia* | 3.4±10.8% | **3.7±11.1%** | **3.6±11.2%** |
| Rhizobiales_Incertae_Sedis | unclassified | 3±2.4% | **2±2%** | **2±2%** |
| Lachnospiraceae | *Epulopiscium* | 2.8±9.2% | 2.6±8% | 2.6±8.1% |
| Fusobacteriaceae | *Cetobacterium* | 2.8±7% | 2.9±7.5% | 2.9±7.6% |
| Burkholderiaceae | unclassified | 2.2±3% | **0** | **0** |
| Enterobacteriaceae | *Escherichia/Shigella* | 2.2±5.1% | **0** | **0** |
| Methylomonaceae | *Methyloglobulus* | 1.8±4.9% | **0** | **0** |
| Pirellulaceae | *Pirellula* | 1.7±1.7% | 1.7±1.7% | 1.7±1.7% |
| Legionellaceae | *Legionella* | 1.6±2.3% | 0.8±1.2% | 0.8±1.1% |
| Chitinophagaceae | *Dinghuibacter* | 1.6±3% | 1±2.1% | 1±2.2% |
| Armatimonadaceae | *Armatimonas* | 1.6±3.2% | 1.2±2.6% | 1.2±2.5% |
| Mycobacteriaceae | *Mycobacterium* | 1.5±7.9% | 1.6±7% | 1.5±7% |
| unclassified | unclassified | 1.5±3.1% | 2.1±3.3% | 1.8±3% |
| Sporichthyaceae | *hgcI_clade* | 1.5±3.7% | 1.1±2.6% | 1.1±2.7% |
| NS11-12_marine_group | *unclassified* | 1.4±2.7% | 1.1±2% | 1.1±2.2% |
| Xiphinematobacteraceae | *Candidatus_Xiphinematobacter* | 1.2±7% | **3.2±14.1%** | **3.2±14.1%** |
| TRA3-20 | unclassified | 1.2±2.4% | 0.7±1.5% | 0.8±1.6% |
| Isosphaeraceae | unclassified | 1.1±1.2% | 1.2±1.1% | 1.1±1.1% |
| Clostridiaceae_1 | *Clostridium_sensu_stricto_1* | 1.1±2.9% | **0** | **0** |
| Hyphomicrobiaceae | *Hyphomicrobium* | 1.1±1.7% | 0.7±1.2% | **0** |
| Nitrospiraceae | *Nitrospira* | 1±2.4% | 0.9±2.1% | 1±2.1% |
| Anaerolineaceae | unclassified | 1±2.8% | 0.9±2.2% | 0.9±2.4% |
| Rubinisphaeraceae | unclassified | 1±1.2% | **0** | **0** |
| Spirosomaceae | *Pseudarcicella* | 1±2.3% | 0.7±1.7% | 0.7±1.7% |
| Chitinophagaceae | unclassified | 0.9±1.7% | 0.8±1.4% | 0.9±1.5% |
