# Supplementary material for: Ranking the biases: The choice of OTUs vs. ASVs in 16S rRNA amplicon data analysis has stronger effects on diversity measures than rarefaction and OTU identity threshold
Source: PLoS One. 2022 Feb 24;17(2):e0264443. doi: 10.1371/journal.pone.0264443 (PMC8870492; doi:10.1371/journal.pone.0264443)
Supplement: Attachment — Submitted filename: letter_reviewers_v2.docx [file pone.0264443.s003.docx]

** Authors’ answers and comments in blue below **

_______________________________________________________________________________

Dear Dr. Chiarello,

Thank you for submitting your manuscript to PLOS ONE. After careful consideration, we feel that it has merit but does not fully meet PLOS ONE’s publication criteria as it currently stands. Therefore, we invite you to submit a revised version of the manuscript that addresses the points raised during the review process.

The reviewer suggests a few, mostly editorial, modifications that should not be too much of a problem. Then the reviewer also suggests the use of a mock community, but does not think it's absolutely necessary. I agree, thus I leave the decision to add results using a mock community to you.

Please submit your revised manuscript by Jan 13 2022 11:59PM. If you will need more time than this to complete your revisions, please reply to this message or contact the journal office at [plosone@plos.org](mailto:plosone@plos.org). When you're ready to submit your revision, log on to https://www.editorialmanager.com/pone/ and select the 'Submissions Needing Revision' folder to locate your manuscript file.

Please include the following items when submitting your revised manuscript:

- A rebuttal letter that responds to each point raised by the academic editor and reviewer(s). You should upload this letter as a separate file labeled 'Response to Reviewers'.
- A marked-up copy of your manuscript that highlights changes made to the original version. You should upload this as a separate file labeled 'Revised Manuscript with Track Changes'.
- An unmarked version of your revised paper without tracked changes. You should upload this as a separate file labeled 'Manuscript'.

If you would like to make changes to your financial disclosure, please include your updated statement in your cover letter. Guidelines for resubmitting your figure files are available below the reviewer comments at the end of this letter.

If applicable, we recommend that you deposit your laboratory protocols in protocols.io to enhance the reproducibility of your results. Protocols.io assigns your protocol its own identifier (DOI) so that it can be cited independently in the future. For instructions see: [https://journals.plos.org/plosone/s/submission-guidelines#loc-laboratory-protocols](https://journals.plos.org/plosone/s/submission-guidelines#loc-laboratory-protocols). Additionally, PLOS ONE offers an option for publishing peer-reviewed Lab Protocol articles, which describe protocols hosted on protocols.io. Read more information on sharing protocols at <https://plos.org/protocols?utm_medium=editorial-email&utm_source=authorletters&utm_campaign=protocols>.

We look forward to receiving your revised manuscript.

Kind regards,

Gabriel Moreno-Hagelsieb
Academic Editor

**Response to reviewer’s comments:**

Reviewer #1: The results provide some helpful considerations for the application and interpretation in 16S amplicon data studies.

The manuscript is presented with a clear structure and language.
The sequencing data underlying the findings will be available upon publication.
The applied statistical analyzes allow answering the problem posed.

Thank you for your time and such positive comments. We have answered these points that you raised below and highlighted the changes in the revised manuscript using the track change option.

1. Is the manuscript technically sound, and do the data support the conclusions?

Here are my comments:
Line 228 "groups across the different analysis treatments was made using Spearman’s signed rank tests."
I am not sure if you mean Spearman rank correlation test or Wilkoxon signed rank test.

We used both tests. The Wilcoxon signed-rank test for repeated measurements tested whether there was a significantly different alpha diversity value on the same samples after distinct sequence processing methods (e.g. Mothur or DADA2). Spearman’s and Pearson’s correlations were then computed to measure the degree of correlation of such measures across methods. We modified the text to clarify this point.

Line 486. The discussion in this paragraph would be clearer if you cite the table/figures showing the corresponding information (correlation and ecological signal).

We agree and have added this information.

Line 509 "samples before rarefaction. In the absence of a mock community, we cannot conclude whether"
What is the reason you did not include a mock community? Although I don't consider this absolutely necessary for the traced objectives, even an in silico mock community would help to gain more insight into the subject.

The idea of this study originated after we confronted the results of our newer assessment of mussel microbiome composition after switching to ASV-based methods, with our previous OTU-based studies. As we observed major differences, we decided to make a more systematic comparison based on our newest set of samples, which resulted in the present manuscript. As this study wasn’t initially planned, we did not include a mock community in our sequencing run, which would have helped identifying potential spurious OTUs. However, given the much higher number of OTUs compared to ASVs, and lower evenness of OTU-based communities, we think that our hypothesis of richness overestimation by Mothur is reasonable, especially as it agrees with previous results based on mock communities (e.g. Prodan et al, 2020, and others cited in MS). We agree that a simulated sequencing run (e.g. using the tool from Richter et al, 2008) that would be processed by both ASV- and OTU-based methods, could help in understanding the finer difference between them, but it would deserve an entirely new study, as it would require (i) a long computation time, and (ii) a tricky calibration as the bacteria in our communities of study generally lack representatives in genome databases.

Therefore, we removed the mention of a mock community within the discussion and simplified the text L520.

Line 168 "... Forward and reverse reads with >2 or >5 estimated errors, respectively,"

Corrected

Line 517 "underlining the sensitivity of OTU-based methods to sequencing errors "
You don’t provide information about the quality statistics from your sequencing data, however this is a central piece of information for both ASV performance and OTU. This information might also be valuable for the discussion.

We added a table with such statistics in Supplementary Table S3. All our samples passed the fastqc standard for sequence quality scores. Globally the statistics were comparable to our previous assessments from environmental samples.

Line 390 and 527. You found differences across both methodologies in some dominant taxa. Did you try to find the reasons behind such differences in your sequence dataset?

We blast-searched the representative sequences of Mollicutes and Verrucomicrobiae over the NCBI database. From the results, our identifications were correct. Our main hypothesis is that differences in sequence processing led to the removal of sequences that were distinct between Mothur and DADA2. It is however difficult to investigate this further without using appropriate mock communities.

References:

Richter, D. C., Ott, F., Auch, A. F., Schmid, R., & Huson, D. H. (2008). MetaSim—a sequencing simulator for genomics and metagenomics. *PloS one*, *3*(10), e3373.

Prodan A, Tremaroli V, Brolin H, Zwinderman AH, Nieuwdorp M, Levin E. Comparing Bioinformatic Pipelines for Microbial 16S rRNA Amplicon Sequencing. Seo J-S, editor. PLOS ONE. 2020 Jan 16;15(1):e0227434.
